# ‘We have no services for you… so you have to make the best out of it’: A qualitative study of Myalgic Encephalomyelitis/Chronic Fatigue Syndrome patients’ dissatisfaction with healthcare services

**DOI:** 10.1111/hex.13900

**Published:** 2023-10-31

**Authors:** Line Melby, Roshan das Nair

**Affiliations:** ^1^ SINTEF, Department of Health Research Trondheim Norway

**Keywords:** dissatisfaction, Myalgic Encephalomyelitis/Chronic Fatigue Syndrome, next of kin, Norway, patients, qualitative study, service delivery

## Abstract

**Introduction:**

People should have access to healthcare services that are effective, safe and secure, patient‐centred, and coordinated and continuous. One group that has consistently reported negative experiences and feels dissatisfied with services are patients with Myalgic Encephalomyelitis (ME)/Chronic Fatigue Syndrome (CFS). The objective of this study was to develop a deeper understanding of the experiences of dissatisfaction among ME/CFS patients and explore the reasons for such dissatisfaction.

**Methods:**

We conducted in‐depth interviews with 48 people from 24 households (comprising patients and family members), providing insight into the experiences of 37 ME/CFS sufferers in Norway. The participants were purposively sampled and included persons of different ages, genders, time since having the condition (3–30 years), and severity.

**Results:**

Four main themes were developed: (1) ‘Nonexistent services’ cover patients’ experience that healthcare services had nothing to offer them after receiving their ME/CFS‐diagnosis. (2) ‘Nonpersonalised services’ documents experiences where patients did receive services, which *in theory* was appropriate for relieving a specific health problem, but *in practice* were experienced as inappropriate because they were not adapted to the patient's need. (3) ‘Slow services’ address patients’ experience of getting services too late (or too little) to be useful. (4) ‘Wrong services’ comprise patients’ experiences of being offered and/or ‘forced’ to accept services that they felt were inappropriate for their health problems.

**Conclusions:**

Providers’ lacking knowledge of the condition and lack of precise recommendations for follow up may partly explain unsatisfactory experiences. Providers’ belief (or disbelief) in the condition could furthermore influence caregiving. Also, systemic issues in the healthcare sector, like high workloads and bureaucracy, can negatively affect care provision. Finally, users’ unsatisfactory experiences may also be due to a lack of patient involvement in the design of such services. Further research should investigate how patients can be involved in service design, and also providers’ perspectives on caregiving and the barriers they experience for providing high‐quality care.

**Patient or Public Contribution:**

The ME‐patient organisation suggested research topics to the call from which this study got funding. Patients and caregivers provided feedback during analysis and interpretation of data.

## 1 INTRODUCTION

Norwegian health authorities define ‘high‐quality healthcare services’ as services that are effective, safe and secure, patient‐centred, coordinated and continuous, available and fairly distributed.^1^ In Norway, to understand and improve healthcare quality, services typically collect Patient‐Reported Experience Measures (PREMs). However, there are limitations in using PREMs (e.g., methodologic issues related to the measurement and interpretation of patient experiences; conflating adequacy with quality of care; confounding factors),^2^ and services need additional in‐depth understanding of service experience when reviewing and re‐commissioning services.

Even though we can learn about service quality from focussing on positive experiences, scholars have argued that we should also investigate negative service experiences, expressed through patients’ accounts of dissatisfaction and disappointment.^3^, ^4^, ^5^, ^6^ Patients can experience satisfaction and dissatisfaction simultaneously,^5^ but understanding patients’ dissatisfaction is seen as particularly important because of its long‐term, negative impact on the patient‐provider relationship and on health‐related behaviours.^5^ Conversely, positive patient experiences contribute to patient engagement, adherence to care plans, and appropriate use of healthcare services.^7^

One group of patients that has consistently reported negative experiences and dissatisfaction with the healthcare services are patients with Myalgic Encephalomyelitis (ME)/Chronic Fatigue Syndrome (CFS).^8^, ^9^, ^10^, ^11^ ME/CFS is a complex, chronic medical condition affecting multiple body systems. Its aetiology is unknown, and there are no biomarkers enabling accurate identification of cases, with diagnosis reached by excluding alternative diagnoses.^12^ The core symptoms of ME/CFS are debilitating fatigue that is worsened by activity; is not caused by excessive cognitive, physical, emotional or social exertion^13^; is not significantly relieved by rest; and postexertional malaise after activity is commonly reported. Patients typically experience sleep disturbances, problems with thinking and concentration, pain, and dizziness. ME/CFS affects patients differently, and is classified as: Mild, moderate, severe, and very severe,^13^, ^14^ largely based on their impairments, abilities to undertake activities of daily living (ADL) and participation in society.

It is difficult to determine prevalence of ME/CFS because different diagnostic criteria have been used, and diagnosis is often delayed by several years because of the heterogeneity of the symptoms and the extensive clinical investigations needed to exclude alternative diagnoses.^12^ The incidence rate is highest in the age groups 10–19 years and 30–39, and the majority affected are women.^15^, ^16^ Precise prevalence rates in Norway are not known.

Norway provides a good, well‐funded healthcare service in comparison with other nationally‐funded healthcare services (e.g., United Kingdom), but despite this, services are limited for people with ME/CFS. In Norway, typically, people with symptoms of ME/CFS see their GP first who then make a referral to specialist healthcare services, which is where a diagnosis is made. Care plans (if any) are made here, but as our study shows, these are not perceived as being adequate or appropriate.

There is no approved treatment for ME/CFS in Norway, so treatment guidelines mainly focus on managing symptoms and how to live with ME/CFS. The United States and United Kingdom have recent comprehensive guidelines, which provide detailed information about the care to be offered.^13^, ^14^ In Norway, however, the ‘current’ (2015) national guideline states: ‘there is no documented standard treatment that can cure CFS/ME’, but acknowledges that there ‘are treatments and strategies that can relieve unpleasant symptoms, contribute to constructive coping and improve patients’ function and quality of life’.^17^ Norwegian service provision typically consists of physiotherapy, cognitive behavioural therapy, and pain relief, but there is no uniform care package.

### 1.1 Understanding patient dissatisfaction

Studies on ME/CFS patients’ experiences with the services have found mixed results, but predominantly address negative and distressing experiences. Three reviews^9^, ^10^, ^11^ summarised patients’ experiences of the diagnostic process and the medical encounters. They found that patients report a high level of distress and dissatisfaction with the quality of medical care, and that they experienced psychological harm,^11^ disruptions in self‐perception, loss of confidence, and identity changes.^9^, ^10^

Studies point to the importance of the relationship between patients and care providers^9^, ^10^, ^11^, ^18^, ^19^, ^20^ for good care experience, and patients may spend much energy finding providers who have knowledge of ME/CFS and whom they can trust.^21^ A relationship breakdown between patients and providers can lead to a lack of empathetic care, and patients may feel helpless and let down,^9^ resulting in them experiencing a highly dissatisfactory service and ultimately disengaging from services.

Norwegian studies about ME/CFS patients’ *experiences* with healthcare services are sparse and have mainly used survey methods. Sommerfelt et al.‘s^22^ internet‐based survey (*n* = 491) found mixed results, with healthcare and social services often described as being insufficient or inadequate, and worsening patients’ symptoms. Participants also reported a lack of knowledge among healthcare providers. However, some found the services provided by occupational therapists and general practitioners to be helpful, but the authors concluded that the services were ‘commonly grossly inadequate’. In another self‐reported questionnaire study^23^ on women members of The Norwegian ME Association (*n* = 431), care quality was rated ‘poor’ or ‘very poor’ by a large proportion in relation to primary care, specialist care, and coordination of care. They concluded that the services had ‘a large potential for improvement’. Yet another study,^24^ using the same sampling method (*n* = 310 women with ME/CFS), explored the perception of ‘continuity of care’ in general practice. They found that almost two‐thirds of participants reported positive experiences across informational, management, and relational continuity dimensions of continuity of care.

We identified only two Norwegian ME/CFS studies that used qualitative research methods. One interview study explored factors perceived as positive or negative among young people with ME/CFS (*n* = 18) in relation to school and everyday life. Participants experienced a lack of educational adaptations and missed social life at school, suggesting a need for school staff to provide more individually‐tailored educational and social adaptations to improve schooling and health among this group.^25^ Another study^26^ focused on the recovery process of two young women with severe ME/CFS. Describing the lived experiences of the women, it emphasised the bodily and everyday life experiences en route to recovery. Experiences with public healthcare services were not really explored, but individually adapted care was described as positive by both participants.

Therefore, based on a limited number of studies, it appears that the Norwegian experience of healthcare services by people with ME/CFS is mixed at best, but largely poor. It is vital that we understand not only *what* patients with ME/CFS are dissatisfied with, but also *why* and *how* such dissatisfaction occurs. In attempting to answer these questions, we examine ME/CFS patients’ and their family members’ perspectives and explore their experiences of dissatisfaction with the healthcare services.

## 2 METHODS

The paper draws on data collected as part of a larger research project,^27^ which combined interviews with survey and register data. The qualitative part of the study was exploratory, and used interviews to obtain insight into ME/CFS‐patients’ and their family members’ experiences with the Norwegian public healthcare services. The design allowed us to be more flexible in our discovery of various aspects of ME/CFS care more generally,^28^ and more specific research questions were developed during the course of the project. Mismatch between patients’ needs and the services’ offers emerged early on in the interviews as a major question for us to explore. The approaches we undertook in designing, conducting, and analysing data from this study were influenced by our interpretative epistemological approach.^29^

### 2.1 Recruitment, sampling strategy and participants

People diagnosed with ME/CFS according to the ICD‐10 manual^30^ (code G93.3) and their relatives were invited to participate. An invitation letter describing the study was distributed to hospitals, the national specialist centre for CFS/ME, etc. where patients might see it, or to the research team's networks. We also posted it on the research project's Facebook site.

Those interested in participating, accessed a link and completed a brief online questionnaire on age, gender, place of living, how long they had been ill, severity of their ME/CFS, and if they were a member of a patient organisation. Of the 230 individuals who wanted to participate, we used maximum variation sampling to select households that were diverse in terms of demographics (age, gender, education, etc.) and ME/CFS features.

In total, we gathered experiences of 37 ME/CFS sufferers through stories from 48 people in 24 households. The households consisted of one or more ME/CFS sufferers of different ages, time since having the condition (3–30 years), and degrees of ME/CFS severity. About half of the households had children under 18 years, and some households had adult children who also had ME/CFS and were living with their parents. Where people were too ill to be interviewed, we interviewed the relatives who cared for them. In some instances, the person with CFS/ME was interviewed together with another household member (e.g., a partner or parent). In some cases, the person with CFS/ME, due to lack of energy or cognitive difficulties, only participated in part of the interview. Table 1 provides an overview of the study participants and Table 2 provides an overview of the demographic characteristics of the persons with ME/CFS.

**Table 1:** Characteristics of study participants.

	Characteristics of the ME/CFS sufferer		Study participants^a^	
Household	Child or adult^b^	Gender	Index person/people with ME/CFS interviewed	Other/Additional informant interviewed
1	Two children	W	No	Mother and father
2	Adult	M	No	Mother and father
3	Adult	W	Yes	
4	Adult	W	Yes	Partner
5	Child	W	Yes	Mother
6	Adult	W	Yes	
7	Adult	W and M	Yes	
8	Two children	W	Yes	Mother and father
9	Two children, one adult	W	Yes	Partner/father
10	Child and Adult	M and W	Ill mother	Father
11	Child and Adult	M and W	Ill mother and father	
12	Child	W	No	Mother
13	Three adults	M, W, W	Ill mother, one ill child	Father
14	Adult	M	No	Partner
15	Child and Adult	W	Ill mother	Father
16	Adult	W	Yes	
17	Child and Adult	M and W	Ill mother	
18	Adult	M	Yes	Partner
19	Adult	W	Yes	
20	Three adults, one child	W, W, W, M	The ill persons	Father
21	Adult	W	Yes	Partner
22	Adult	M	Yes	
23	Adult	W	Yes	Partner
24	Adult	W	Yes	

Abbreviations: CFS, Chronic Fatigue Syndrome; M, man; ME, Myalgic Encephalomyelitis; W, woman.

a For each household, we interviewed someone with ME/CFS and/or a significant other. We have indicated where the person/people with ME/CFS in the household participated in the interview (indicated as ‘Yes/No’), and where we had another member of the family (without ME/CFS) who was interviewed (either in addition to or instead of the person with ME/CFS). In the ‘Other/Additional informant interviewed’ column, we specify the relationship of this informant to the index person with ME/CFS.

b Where someone is labelled as a ‘child’ they are under the age of 18.

**Table 2:** Group level demographics of people with Myalgic Encephalomyelitis/Chronic Fatigue Syndrome.

	Women	Men	Total	Age range	Average age
Adults	16	8	24	19–59	42,7
Children	9	4	13	10–17	14,1
*Total*	25	12	37		

### 2.2 Data collection

Before the interviews, we developed an interview guide with five focus areas, with prompts to explore that area (see Table 3).

**Table 3:** The five focus areas for the interviews and the questions that probe these areas.

Focus area	Prompts
The development of the condition and the route to diagnosis	When did you get ill, and how did the condition develop?
	What was the route to a diagnosis like?
	How was the household affected during this first phase of having ME/CFS?
Experiences with public health and welfare services (healthcare, social services, schools)	What kind of services have you been in contact with, the frequency of contact, and satisfaction with the encounters?
	How is the household affected from the ME/CFS currently (at the point of interview)?
Engagement in patient organisations, peer groups, etc.	Are you a member of a patient organisation or informal peer group/support group?
	Why or why not?
Satisfaction with services	What services/types of help have you been most satisfied with?
	How can unsatisfactory services be improved?
Positive experiences during the illness period	What positive experiences would you highlight from the period you have been ill?
	What brings hope to you?

Abbreviations: CFS, Chronic Fatigue Syndrome; ME, Myalgic Encephalomyelitis.

In interviews, although we were mainly interested in unpicking reasons for satisfaction and/or dissatisfaction with services, we aimed to obtain an in‐depth understanding of the participants’ experiences with being ill and their encounters with the health and welfare services more generally. Interviews were conducted by seven researchers between June and November 2019. The interviewers were five women and two men, who were sociologists, a political scientist, and a healthcare services designer. They had a Masters or PhD level education. In about half of the interviews, two researchers participated, and the rest were conducted by one of the researchers. The interviewers did not personally know the participants. All researchers had experience conducting interviews and interviewing vulnerable groups.

All interviews were conducted in participants’ homes, in Norwegian, and audio‐recorded and transcribed verbatim by research assistants. Field notes provided additional context for the interview data. Interviews lasted between 40 and 150 min. The quotations used in the Results section have been translated from Norwegian.

### 2.3 Analysis

We used an inductive thematic approach^31^ for data analysis as part of a reflexive thematic analysis.^32^ The first author read through all the transcripts and searched for statements describing various experiences of dissatisfaction (Phase 1, familiarising with the material), and then systematically coded the material. Excel was used to organise the material and the codes. The initial coding led to 20 codes. Some of these overlapped, one needed to be divided, and some only contained a few instances. One example was the large code ‘mismatch between patients’ needs and services offered, which later was divided into the themes ‘no services is offered’, ‘no individual adaptation’, ‘wrong service’. After a review of the codes, four main themes were developed and discussed with the second author. The names of the themes, and their content (subthemes) were revised through discussions between the authors, establishing the final four themes.

Based on our interpretations of the data, we have organised the themes to represent possible reasons for dissatisfaction with the services among ME/CFS sufferers and their relatives. They are not mutually exclusive, and one experience may therefore be characterised by more than one theme. However, when presenting examples from each theme, we have extracted the parts of an experience that clearly depict the theme. We report the results as per the consolidated criteria for reporting qualitative research.^33^

The study team engaged in critical self‐reflection during study team meetings where progress on the interviews and analysis were discussed. Issues the study team were grappling with in terms of the participants’ stories/narratives were discussed to raise our own awareness of how our own perspectives could be influencing our analyses.

## 3 RESULTS

The analysis resulted in four main themes of service experiences that was associated with study participants’ dissatisfaction with the services. Table 4 provides an overview of themes and sub‐themes.

**Table 4:** Themes and subthemes.

Outcome/experience	Main theme	Subthemes
Dissatisfaction, disappointment, distress	Nonexistent services	Lack of treatment recommended by Norwegian health authorities Lack of practical help Lack of information about managing the condition
	Nonpersonalised services	Help was offered, but not adapted to CFS/ME patients’ specific needs
	Slow services	Information about coping with the condition comes too late Services are offered too late to be useful
	Wrong services	Engagement with services is mandatory and conditional on receiving social and labour benefits Services offered do not address your problem

Abbreviations: CFS, Chronic Fatigue Syndrome; ME, Myalgic Encephalomyelitis.

### 3.1 Nonexistent services

Once they had received their ME/CFS‐diagnosis, patients were told by healthcare providers that there was no effective treatment, and they consequently felt abandoned and left to understand and deal with their diagnosis by themselves:

A hospital physician called me and said: ‘You have ME. We have no services for you currently, so you have to make the best out of it’. This was in 2011. (…) I tried to educate myself; what is ME? I did not have a clue. There were not many specialists or physicians who could tell me. I went online. (W21)

Another participant had a similar experience. After being informed about the diagnosis, she felt alone and was unsure what to do:

…there was no network around me. When you have received the ME/CFS diagnosis, [the hospital said], then you have to manage on your own because we cannot help you anymore. So, then I sat there and wondered, what is the right thing to do now? Because inside me, I still had this thought that exercise was good and that you should live a life that gave increased health benefits. But I still had a long way to go to learn that it did not fit with the ME/CFS diagnosis. (W15)

Alongside lack of treatment, there was a lack of information about ME/CFS, for example, how to act to prevent the condition from getting worse. Many had acted on ‘common sense’ when they felt tired, like getting fresh air, exercising, and trying not to dwell upon the condition. Many reported having met with healthcare providers who had little knowledge of ME/CFS, and therefore did not get qualified advice on how to act. Furthermore, all had encountered providers who opined that ME/CFS was a psychosomatic disease, an opinion not shared by the participants, and several participants had to provide information about ME/CFS to the services. However, most of them thought it was of little use. Providers’ knowledge gaps of ME/CFS and symptom management also led to several instances where patients had to take considerable responsibility for their own health.

Since there are no medicines that can help persons with ME/CFS, you kind of get ignored. You could say that it is a natural consequence of not having sufficient medical solutions. So, what can you do? But it also means that ME/CFS patients (…) must take much more responsibility for our own health. (W22)

Patients encountered a lack of services in several phases in their ME/CFS journey. Previous examples illustrate encounters with healthcare services immediately after being diagnosed. Others who had been ill for a long time (postdiagnosis) talked about the lack of practical help in ADLs, like assistance in shovelling snow, gardening, and grocery shopping. A couple, both with ME/CFS, had tried to buy practical help from a private provider, because the user fee/co‐payment for the public service was higher than what they paid the private provider.

Obviously, we would have benefited from having some practical help in the house. We have tried it, that was a private initiative. But we also get tired from having help. (W7)

Practical help and assistance of various kinds being ‘tiresome’ was a shared experience across many participants. They referred to experiences with getting help characterised by lack of respect and distrust from the providers, and their needs not attended to. There were examples of people who had been ill for many years, who now intentionally avoided any contact with healthcare services. They felt that contact with healthcare workers, even if only for a checkup, would make them worse.

In summary, ME/CFS participants faced a lack of clinical care, information, advice, and practical health on how to look after their own health. This made them feel abandoned and left to care for themselves, coping with a disabling condition. This, in turn, led several participants to turn to complimentary and/or experimental medicine, and/or to a withdrawal from social and public life and healthcare services altogether.

### 3.2 Nonpersonalised services

Some participants did receive services, which *in theory* were targeted and appropriate for relieving a specific (health) problem. This included services like home healthcare, follow‐up of health problems *other than* ME/CFS, and practical home help (like cleaning). However, *in practice*, since these services were not adapted to patients’ specific needs, they were experienced as inappropriate and even damaging, making patients more ill. For example, ME/CFS‐patients are typically easily exhausted, and most are sensitive to light, sounds, noise, smells, and so forth, and yet a common experience was that the services did not sufficiently pay attention to such challenges. One participant talked about her experience when being admitted to hospital:

When I came into the Emergency room, the standard examinations were done there. But I was put in the corridor, under the pretext that I should be observed. But to lie in the corridor when you are so sick, that is the exact opposite of what you need. Illuminated hallway, lots of noise. Noise, light, everything. (W19)

Another example came from a mother with a bedbound daughter:

We were granted home healthcare [because she was not able to go to the GP], including having blood tests taken. But she [the daughter] has said she won't have home healthcare anymore, because the nurses are very poor at inserting the needle. The service has been introduced as a good [measure], but they enter the room kind of like clowns and are trying to make her laugh. And then she is lying there and is seriously ill and does not have the energy to meet them. (…) We have given them plenty of information [about her condition], but it doesn't seem to go in. They have to turn on the lights … so [in her room] we have removed the light bulbs just to prevent the floodlights being turned on. Now, no one can turn on lights we can't cope with. (W15)

There were several examples of healthcare workers entering patients’ homes and, according to our participants, acting insensitively, and not considering the specific needs of the person with ME/CFS. Participants mainly described this from a perspective where they considered healthcare workers lacking knowledge about the condition and how to act in an ill person's presence. Trying to educate home healthcare workers* on how to behave did not appear to alter their behaviour. This consequently led to patients feeling they had no other option but to terminate services, like home healthcare services. Therefore, it meant that severely ill people were left without any service at all.

Patients can opt out of having some services, like home healthcare. Other services are mandatory or emergencies, where they cannot opt out. Several participants had experiences with the labour and welfare office (NAV), which is a mandatory contact point when a person still has some relation to the labour market, for example, before being eligible to receive disability pension. One participant explained that meetings with NAV were not adapted to his needs, and having to wait outside the office building felt both physically and mentally challenging:

It is these system‐made routines they [NAV] have (…) Many of the meetings were scheduled for 9 in the morning. The office building was closed, so I could not get into the waiting room. And I experienced it to be very stigmatising to have to wait outside. In addition, I had a significant drop in blood pressure, orthostatic intolerance as it is called. It means I just have to sit down. (M14)

To summarise, all participants had experience of some or most services not being adapted to their needs. This led to patients either refusing to receive the service or terminating nonmandatory services. For others, it led to pushing themselves too much, enduring difficult situations, and getting more ill as a result.

### 3.3 Slow services

Some participants received services ‘too late’ in the ME/CFS trajectory to be useful, or the ‘amount’ of services provided being ‘too little’. Participants talked about under‐staffed publicly funded services that were experienced as bureaucratic and slow. When they eventually were granted access to a service (e.g., receiving information about how to cope with ME/CFS, managing their energy, or having an assistant to help with ADL), participants felt that their health condition had deteriorated. Consequently, they did not benefit from the service as much they would have had they received the service earlier:

What if I had gotten an assistant while I still could be a bit up? But all the help I have gotten has been given to me after I became too sick. If I had had the assistant earlier, I might have been able to keep up my functioning. Maybe, if I could have had a some more help, I would not have ended up where I am now. Because I am convinced that I pushed myself too much… because I wanted to continue taking care of our home. (W15)

The same participant had also experienced that technical aids like crutches and wheelchairs were offered much later than when she would have benefitted from them.

Even though I had a GP who wasn't very collaborative, the occupational therapy service was. That was very good. But everything came too late. I got crutches when I needed a wheelchair, and I got a wheelchair when I needed a (special) bed. (W15)

A third example of a service that delivered too late for it to have been useful was where a child was sick and could not attend school, and her parents tried to organise help for her through a ‘school robot’ so she could follow the teaching from home when she was fit for it. The robot, placed in the classroom, would function as a communication gateway between the school and the pupil at home. However, it turned out to be complicated to organise the service, even though it is frequently used throughout Norway for other user groups. Her mother said:

It was [issues] with the Telecom provider and Wi‐Fi, it did not work, and it did not work out. And who pursues it from the school? I tried to ask a school counsellor and several others, but they did not know. So, then I was sending several letters and [talking to] the parent‐school collaboration group and everything. And in the meantime, the girl became actually too sick to benefit from it. (W8)

In summary, we found several examples of patients and their families getting services too late in the course of their ME/CFS for these services to be useful. When talking about such incidents, participants appeared less angry and upset than when talking about the lack of personalisation of services, but expressed more of a sense of resignation over the ‘system’ working slowly and being bureaucratic.

### 3.4 Wrong services

Some participants were offered and/or ‘forced’ to accept what we refer to as a ‘wrong service’. It is important to clarify that we are not advocating or arguing for or against any specific form of treatment or service, but only reporting what our participants felt was the ‘wrong service’ *for them*. Many participants had experienced being enrolled in rehabilitation or treatments that they felt did not respond to their problems. Participants were particularly critical of various cognitive therapies and treatments that were not developed for people with ME/CFS. One participant discussed being offered cognitive therapy online by her GP:

Cognitive therapy. I said, all right, I'll try that (…) My physician gave me the link to an online course, based on cognitive therapy. (…) it ended with me writing a furious feedback comment, because the whole course was built on: ‘do you have negative thoughts, and then have heavy emotions afterwards?’. It was the recipe for everything. (…) I have a limitation caused by my physical health. It exists here and now, it is nothing I have thought. It is now, it is all the time, and it has been there for several years. It is nothing mental. (M22)

Besides critically questioning different types of cognitive therapies because they felt they did not address their problems, our participants also called for concrete help with physical problems. One woman remembered her stay at a rehabilitation clinic:

During the four weeks we covered many topics. I was often too sick to participate, but I got it on paper so I have a full overview of the topics they went through. Not one hour, not one minute was devoted to symptom relieving treatment and there were people there who are struggling and had not slept for several years, who had not received any painkillers. They could really benefit from being aware that there is something called ‘symptom relief’. But that was not mentioned at all; I do not understand it. (W6)

Some of the examples in the ‘wrong service’ category were services that were well‐intended but did not match the patients’ needs or expectations. However, there were also examples of ‘services’, or rather activities, that were mandatory for our patient participants to be considered eligible for disability pension. These activities, typically work assessment activities, or rehabilitation programmes, were looked upon unfavourably (or ‘wrong’) by participants. Participants felt, in general, that these forced‐upon‐activities did not help them at all, and that attending them in fact made them even sicker. One participant talked about her efforts before being granted disability pension.

[Staff member at NAV] said that he had to sign me up for a job clarification programme to assess my residual work ability, and ‘it's just something you have to do’. I told him that I knew others who were at my level [of health] who had not had to. (…) But he was absolutely adamant that I had to. (…) I got worse and worse from these things. I took notes along the way and have written a letter to NAV afterwards, I spent two and a half years on that letter. I sent it about a year ago. I told them how I experienced the job training, how I felt along the way, how it has affected me after‐ I deteriorated after that experience and it [my symptoms] has never improved. (W10)

In summary, participants had experienced services and measures that they felt did not address their ME/CFS. Some of these issues seem to stem from a lack of knowledge of what works, as well as disagreement on how ME/CFS should be managed and patients followed up. The result for many of our patient participants was that they felt sicker after participating in the services than before.

## 4 DISCUSSION

Previous studies suggest that high‐quality services are characterised by being effective, safe and secure, patient‐centred, coordinated and continuous, utilise the available resources, and are available and fairly distributed.^1^ Our study shows that ME/CFS patients do not experience healthcare and welfare services to meet such quality criteria. Our analysis has focused on ME/CFS‐patients’ disappointments, distress and dissatisfaction with the healthcare and welfare services, and has sought to gain a better understanding of what lies beneath the dissatisfaction.

Patient satisfaction is an ‘undertheorized concept’,^34^ and experts warn of the ‘noncritical utilisation’ of marketing theories of ‘customer satisfaction’ to understand patient satisfaction,^35^ and we would add, particularly in a publicly funded healthcare system. Therefore, while there is no single or universally accepted theory of patient satisfaction, any attempt of theorising this construct must be multidimensional.^35^ In this discussion, therefore, we revisit each theme in relation to extant literature, sociopolitical and healthcare contexts, and not in terms of a specific theory. However, we acknowledge that others have used specific theories (e.g., epistemic injustice) in their analysis of data.^36^, ^37^

ME/CFS as a physical health condition has courted controversy due to different conceptualisations of ME/CFS among patients, healthcare professionals, and scientists.^36^, ^38^ The controversy has evolved around the cause, and consequently, what kind of treatment and follow up patients should be offered. Currently, there is a lack of evidence‐based treatments. The Norwegian national guideline for ME/CFS^17^ presents different care/management strategies, but emphasises that individually‐adapted care is necessary.^17^ Therefore, while there is no single blueprint for ME/CFS services, such guidelines are important because in publicly funded healthcare systems, like in Norway, services are delivered and assessed based on the extent to which they adhere to these guidelines. The requirement for personalisation of care is positive but presents challenges for healthcare workers in determining what kind of care to offer patients. The lack of precise recommendations may lead to uncertainty among healthcare workers when it comes to how to act.^39^ They might, for example, stick to one treatment recommendation, without sufficiently considering the person's individual needs. Our study shows numerous examples of patients having to attend the same courses, like cognitive therapies or job clarification programmes, even though they describe them as ‘wrong services’ for them, which suggests that personalisation does not always occur.

Another related explanation for participants’ dissatisfaction with the services appears to stem from providers lacking knowledge of the condition. A review and meta‐synthesis of qualitative studies on ME/CFS^10^ identified several studies that found a lack of knowledge among healthcare providers about the condition and follow up recommendations. One study found that primary care physicians used social and cultural knowledge (insight gained from the media and the Internet and through observations of patients outside the clinical setting) instead of biomedical knowledge in diagnosing ME/CFS.^40^ Indeed, several studies have recommended better training and educational initiatives for physicians^10^, ^41^ and for school personnel.^25^ Our participants gave many examples of encounters with healthcare workers where they thought that the professionals had little knowledge of ME/CFS, and that their attempts to inform and educate them, in many cases, was futile.

Such lack of knowledge could also create challenges to delivering personalised services to the individual ME/CFS sufferer; delivering personalised care being seen as a top priority for the Norwegian healthcare service. Participants gave several examples of services that were not adapted to their health condition and to their needs. Based on our findings, we cannot say for certain whether services were poorly adapted due to service providers lacking knowledge, but it appears that patients’ *perceptions* of service providers lacking knowledge is what drives the dissatisfaction.

Dissatisfaction with the services, and particularly experiences of nonpersonalised services and slow services, could result from systemic issues also; for example, the organisation of healthcare and welfare services, as noted by the participants. Public services are frequently characterised by high workloads and bureaucracy, and require patients to negotiate complex organisational systems.^42^ This could prevent staff from making the needed adaptations *and* keeping up the tempo in service delivery, for example in the cases where ME/CFS sufferers received technical aids too late for them to be useful. Patients’ needs for adaptations require considerable flexibility from services. A UK study on ME/CFS patients’ encounters with specialist healthcare services found that good accessibility and flexibility of services contributed to positive service experiences. For example, being offered appointments late in the day was seen as positive, because this was adapted to the patient's circadian rhythm.^8^ We have not found many other studies that delve into ME/CFS patients’ various needs for personalisation of services, however, it seems obvious that the contrast between the unpredictable and fluctuating nature of ME/CFS and the services’ need for planning and predictability leads to some challenges in personalised service delivery.

Another explanation for both the lack of personalised services and for ‘wrong services’ could be related to providers’ belief, or rather disbelief, in the condition. Again, this could be related to staff lacking knowledge of ME/CFS. The lack of belief among providers that ME/CFS is a ‘real’ condition has been reported before. Patients report being met with suspicion, scepticism, and disbelief from healthcare workers,^9^, ^10^, ^37^ and patients may not agree with healthcare workers’ conceptualisation of ME/CFS or what they think are effective treatments.^37^ Because most ME/CFS symptoms are ‘invisible’, family members can also have problems accepting the condition.^43^ Distrust between providers and ME/CFS sufferers and their families runs like a red thread throughout our study. Only patients and family members were included in our study, and consequently we do not have providers’ views on the condition from their own accounts. However, based on participants’ descriptions of their encounters with the services, it appears that many providers do not believe that ME/CFS is a ‘real’ condition. This can affect their views on (and practice of) how to treat or care for patients. Furthermore, being met with disbelief and scepticism in the medical encounter also affects patients’ behaviour, for example, deciding to leave a service. Problematic clinical interactions are not uncommon and found in several studies,^11^ and can arguably lead to poor service offers.

A final explanation for unsatisfactory experiences among patients may be a lack of patient involvement in the design of such services. Patient involvement is known to improve quality of care and patient experiences, among other things.^44^, ^45^, ^46^ Our participants reported several instances where they experienced little or no participation in decisions over their own health. Lacking patient participation in studies can be caused by patients not being invited to participate, and it can be complicated by patients being very ill. Several studies, summarised in a systematic review,^45^ point to ME/CFS severity as a barrier to patient participation, and good health being seen as a facilitator. The same review describes that systematic patient involvement is not widespread in clinical practice, that most healthcare providers are not trained in shared decision‐making, and therefore lack knowledge of how to involve patients. This can be a possible reason for ME/CFS patients’ experiences of services and healthcare professionals not involving them in their care. Furthermore, an essential prerequisite for patient involvement is a trusting patient‐doctor relationship.^45^ Our results show that many patients do not trust their physicians, and that the relationship between patients and many providers is instead characterised by distrust and suspicion. Finally, several participants talked about their decision to withdraw from the services because they felt the services had nothing to offer that made them better. Such feelings will again discourage patient involvement.

The literature on patient dissatisfaction points both to reasons for dissatisfaction and how dissatisfaction manifests. Lack of access and availability of services, bureaucratic procedures, poor communication, and challenging relationships between providers and patients can lead to dissatisfaction.^4^, ^5^, ^6^, ^9^, ^10^, ^11^, ^47^, ^48^ Our findings are in line with this. In particular, instances of poor service encounters and challenging relationships (as experienced by participants) were found. We have, for example, shown that participants experience being disempowered and devalued (cf. (5)), and not being seen and their concerns affirmed.^47^ An explicit focus on these matters would have given a more detailed picture of such experiences, but that was outside the scope of this paper, and could be a focus area for future research.

### 4.1 Strengths and limitations

Our study has several strengths in terms of the diversity of our participant pool and including significant others. Unlike in a survey, our data, obtained through interviews were rich and in‐depth, offering considerable nuance. Indeed, few Norwegian studies have used qualitative methods to address dissatisfaction with ME/CFS services, so our analyses offer a unique insight into this issue. We had several interviewers, which allowed us to collect data by people without having a single viewpoint, agenda, or belief being promulgated during the interviews. However, this also posited a challenge as to how quality and consistency could be maintained. Therefore, all interviewers used the same interview schedule and were trained by the lead author to undertake the interview. Also, although not formally analysed, we did not observe major differences in the quantity, quality or ‘tone’ of the data obtained from the different interviewers. There are some limitations, however, that we wish to acknowledge. First, we only recruited and interviewed patients who were ill – none who had recovered/improved. People with ME/CFS who have recovered might have other perspectives and complimentary insights and shine light on other aspects of the patient journey. Second, we only involved people with ME/CFS and their significant others, and service providers were not interviewed. Therefore, we were not able to compare the views of those who received the services and those who provided them directly. This would have been interesting to obtain a more comprehensive view of the problems and potential solutions.

### 4.2 Conclusion

Nonexistent services, nonpersonalised services, slow services, and ‘wrong’ services are four key reasons as to why ME/CFS patients and their family members experience distress and dissatisfaction with the health and welfare services in Norway. It is important to highlight that patients also do describe good experiences with the services, so the picture is not entirely bleak. But, by shedding light on the negative experiences, we hope to contribute with knowledge that can be used for improving services. Further work in the field of service design for ME/CFS patients should focus on increasing knowledge among care providers and developing a more patient centred care model. Further research should investigate providers’ perspectives on caregiving, and the barriers they experience for providing high‐quality care. Providing ongoing care to patients and households affected by ME/CFS is a public responsibility, so we cannot continue a practice where patients are not offered appropriate, personalised services, or they have to choose to discontinue with services because the services are experienced as more distressful than helpful.

## AUTHOR CONTRIBUTIONS

*Conception and Design*: Line Melby. *Interpretation of data and Writing the manuscript*: Line Melby and Roshan das Nair. Both authors read and approved the final manuscript.

## CONFLICT OF INTEREST STATEMENT

Roshan das Nair has no declarations in relation to this study, but has received funds from Biogen, Novartis, and Merck (speakers bureau) to deliver lectures on psychological and neuropsychological aspects of multiple sclerosis. Line Melby declares no conflict of interest.

## ETHICS STATEMENT

The project was approved by the Regional Committee for Medical and Health Research Ethics Central Norway, ID 7345 and application number 2018/293, and performed in accordance with the Declaration of Helsinki. All participants were fully informed about the study and provided their written consent for participation in interviews.

## Data Availability

The data set used for the current study is not publicly available to protect the anonymity of the participants, but are available from the corresponding author on reasonable request.

## ENDNOTE

* In Norway, ‘home healthcare workers’ are both nurses and other healthcare workers who work in the municipality and provide care in people's homes.
